# Effects of Soothing Liver and Invigorating Spleen Recipes on the IKKβ-NF-κB Signaling Pathway in Kupffer Cells of Nonalcoholic Steatohepatitis Rats

**DOI:** 10.1155/2015/687690

**Published:** 2015-10-04

**Authors:** Xiang-Wen Gong, Yong-Jian Xu, Qin-He Yang, Yin-Ji Liang, Yu-Pei Zhang, Guan-Long Wang, Yuan-Yuan Li

**Affiliations:** Department of Traditional Chinese Medicine, Medical College of Jinan University, 601 Huangpu Road West, Guangzhou, Guangdong 510632, China

## Abstract

This study investigates the effect of soothing liver and invigorating spleen recipes on steatohepatitis examining the IKK*β*-NF-*κ*B signaling pathway in KCs of NASH rats. SD male rats were randomly divided into 8 groups, and the NASH model was induced by a high-fat diet (HFD). After 26 weeks, liver tissue was examined in H&E stained sections and liver function was monitored biochemically. KCs were isolated by Seglen's method, with some modifications. The mRNA and protein expression of the IKK*β*-NF-*κ*B signaling pathway components was examined by quantitative PCR and Western blotting. The results show that the high-fat diet induced NASH in the rats, and the soothing liver recipe and invigorating spleen recipe decreased the levels of TNF-*α*, IL-1, and IL-6 in KCs, as well as inhibiting the mRNA and protein expression of the IKK*β*-NF-*κ*B signaling pathway components. In conclusion, the experiment indicated the importance of the IKK*β*-NF-*κ*B signaling pathway in KCs for the anti-inflammatory effects of the soothing liver and invigorating spleen recipes.

## 1. Introduction

Nonalcoholic fatty liver disease (NAFLD) encompasses a spectrum of diseases ranging from simple hepatic steatosis to the aggressive condition, nonalcoholic steatohepatitis, whereas NASH is an aggressive liver disease which leads to advanced fibrosis, cirrhosis, and even hepatic failure [1]. NAFLD is also a strong independent predictor of cardiovascular disease and may play a central role in the cardiovascular risks of the metabolic syndrome [2]. Epidemiological studies suggest that NAFLD has become the most common cause of chronic liver disease worldwide among children and adolescents, with a prevalence in the west of approximately 15~35% [3]. The overall prevalence of NAFLD in China was 20.09% (17.95~22.31%) [4]. Hence, studies of the prevention and treatment of NAFLD are important.

Because the pathogenesis of NAFLD is incompletely characterized, there is no consensus regarding the most effective and appropriate pharmacologic therapy [5]. Herbal treatment for NAFLD has received increasing attention in recent decades due to its wide availability, minimal side effects, proven therapeutic mechanisms, and benefits [6]. Evidence-based medicine supports this point of view [7]. Based on TCM theory, liver stagnation and spleen deficiency were principal pathogenesis of NAFLD. Following the principles of “prescription-syndrome correspondence,” the important treatment would be soothing liver and invigorating spleen recipes [8]. Clinical and experiment studies showed that soothing liver and invigorating spleen recipes could effectively treat NAFLD [9, 10].

The soothing liver recipe (Chaihu-Shugan-San) is a classical formula from “Jingyue Quanshu” that was written by Jingyue Zhang, a famous Ming dynasty physician, in China in 1640 A.D. The invigorating spleen recipe (Shen-ling-bai-zhu-San) is also a famous classical formula recorded in “Taiping Huimin Heji Ju Fang” that was written by the Imperial Medical Bureau of the Song Dynasty in 1078 A.D. The invigorating spleen recipe is recognized in the widely used official Chinese Pharmacopoeia. According to TCM theory, the soothing liver recipe dredges liver qi and enhances blood circulation and is prescribed mainly for liver qi stasis. The invigorating spleen recipe invigorates the spleen and stomach qi, which it is mainly used to restore. Chaihu-Shugan-San and Shen-ling-bai-zhu-San have a significant effect on dredging by the liver and spleen deficiency.

IKK*β*-NF-*κ*B is an important redox-sensitive and proinflammatory transcription factor that plays a critical role in the regulation of a variety of genes important in cellular responses [11]. Our previous study showed that rats fed a high-fat diet for 16 weeks developed NAFLD and that I kappa B kinase *β* (IKK*β*), phospho-IKK*β* (p-IKK*β*), and nuclear factor-*κ*B (NF-*κ*B) were highly expressed in their KCs, implying a relationship between NAFLD and the IKK*β*-NF-*κ*B pathway [12]. We next asked whether rats fed a HFD for 26 weeks would develop NASH and, in this paper, we study the effects of soothing liver and invigorating spleen recipes on inflammatory markers and proteins involved in IKK*β*-NF-*κ*B p65 pathway in KCs to explore some of the underlying mechanisms involved. To explore the evolution of the pathology, we also examined the inflammatory changes that accompany NAFLD at different periods.

## 2. Materials and Methods

### 2.1. Preparation of Soothing Liver and Invigorating Spleen Recipes

The soothing liver recipe is composed of seven Chinese herbs: Bupleuri radix, Citri reticulatae pericarpium, Chuanxiong Rhizoma, Cyperi Rhizoma, Bitter Orange Aurantii Fructus, Paeonia lactiflora Pall, and Glycyrrhizae radix, in a traditional dose ratio of 6 : 6 : 5 : 5 : 5 : 5 : 3. The invigorating spleen recipe includes Dolichos lablab, Atractylodes macrocephala Koidz, Poria cocos (Schw.) Wolf, Glycyrrhizae radix, Platycodi radix, Sulphur, Panax ginseng C. A. Mey, Amomum villosum Lour, Dioscorea opposita Thunb, and Semen Coicisin a ratio of 5 : 5 : 5 : 5 : 4 : 3 : 3 : 3 : 2 : 2. The integrated recipe is a mixture of CSGS and SLBZ in a 1 : 1 ratio. All Chinese medicines were purchased as formula granules from Shenzhen Sanjiu Medical Co., Ltd. (1005001S). The formula granules were dissolved in distilled water and stored at −4°C in a refrigerator.

### 2.2. Animals

120 SD rats (6 weeks old, 200 g ± 20 g) were obtained from the Laboratory Animal Research Center of Guangzhou University of Traditional Chinese Medicine (China, Animal License Key no. 0107792; License no. SCXK (Yue) 2008–0020). The use of animals in this study was approved by the Ethics Committee of Medical College of Jinan University. The rats were separately housed in the Animal Administration Laboratory, Jinan University, at a controlled temperature (24°C ± 2°C) and humidity (70%  ±  10%) with a 12 h light and 12 h dark cycle, with free access to food and water.

### 2.3. Grouping and Modeling

After 7 d for adaptation, the rats were randomly divided into 8 groups, each of 15 rats (liver samples were taken from 9 rats of each group and KCs were isolated from the remaining 6). The groups comprised normal, model, low-dose soothing liver recipe (L-SLG), high-dose soothing liver recipe (H-SLG), low-dose invigorating spleen recipe (L-ISG), high-dose invigorating spleen recipe (H-ISG), low-dose integrated recipe (L-IG), and high-dose integrated recipe (H-IG) group. NASH was reproduced in our rats by our previously reported method [13] with some minor modifications. The normal group of rats had free access to a normal chow diet, and the model group of rats were fed a HFD composed of regular chow 88%, axungia porci 10%, cholesterol 1.5%, and bile salt 0.5%. All recipes were given by gastrogavage [14] at 8 am at 1 mL/100 g body weight for the low-dose groups (equivalent to the human dose) and 3 times this volume for the high-dose groups. Rats in the normal and model groups were fed with the same dose of distilled water once daily at 8:00 am. All treatments lasted for 26 weeks.

### 2.4. Histopathological Examination of Liver Tissue

Rats were anesthetized by intraperitoneal injection of 3% pentobarbital (0.2 mL/100 g body weight) and a portion (approximately 1 cm × 1 cm × 0.5 cm) of liver approximately 0.5 cm from the edge of the right hepatic lobule was removed and paraffin-embedded for sectioning and hematoxylin-eosin (H&E) staining. The steatosis, fibrosis, and inflammation of NASH were identified by light microscopy.

### 2.5. Biochemical Test in Serum

Liver tissues were placed in isopropanol and homogenized with a TissueLyser-II homogenizer, centrifuged at 3000 ×g, 4°C for 10 min, when the clear supernatants were collected. Levels of alanine aminotransferase (ALT), aspartate aminotransferase (AST), and the AST/ALT ratio in the serum were determined with automatic biochemical analyzer.

### 2.6. Separation and Identification of KCs

KCs were isolated and identified from 6 rats in each group as previously described [15], and some modifications were made. After rats were anesthetized, the liver was perfused in situ with 200 mL 0.5 mmol/L Ethylene Glycol Tetraacetic Acid (EGTA) in D-Hanks at 20 mL/min, 37°C, until the color of the liver changed into amber. Then the liver was transferred to a culture dish and was perfused ex situ with 0.03% collagenase IV in Hanks, which contained 5 mmol/L calcium ions and was preheated to 37°C, at 20 mL/min in a recirculating fashion for 15 min. The liver was then placed in 10 mL RPMI-1640 culture medium containing 10% fetal calf serum (FBS). The capsule and fibrous tissue were removed, and the remaining tissue was cut into small pieces. After the obtained liver homogenate was filtered through a 200 *μ*m and 300 *μ*m nylon mesh, the cell suspension was centrifuged at 350 rpm, 4°C, for 3 min and clear supernatant was collected in another tube and centrifuged at 1050 rpm, 4°C, for 10 min. The cell pellet was subsequently resuspended in RPMI-1640 containing 10% FBS.

Then some 15 mL centrifuge tubes were carefully laid into 2.5 mL 24% Nycodenz working solution at the bottom, 2.5 mL 11% Nycodenz working solution in the middle layer, and 2.5 mL the cell suspension at the top. Then it was centrifuged at 2500 rpm, 4°C, for 15 min. KCs cloud appearance between the 11% Nycodenz layer and 24% Nycodenz layer was collected into another 15 mL tube, resuspended in GBSS, and then centrifuged twice at 1050 rpm, 4°C, for 15 min. The cell pellet was then resuspended and seeded on a culture dish at a density of 2–5 × 10^6^ cells/mL with RPMI-1640 containing 10% FBS and incubated in a 5% CO_2_ atmosphere for 30 min at 37°C. By further using adhesion purification, KC purity was improved, and cell viability was tested by trypan blue dye exclusion.

### 2.7. Determination of Inflammatory Cytokines in KCs

KCs from each group were isolated and identified. The cells were then centrifuged, washed 3 times in PBS, adjusted to 1 × 10^6^/mL, and sonicated at 4°C for 15 min at 10000 rpm. Clear supernatants were used for the cytokine measurements. TNF-*α*, IL-1, and IL-6 were measured by enzyme-linked immunosorbent assay (ELISA) using ShangHai ExCell Biotechnology Co., Ltd. (China) kits: 24122012-002 for TNF-*α*, ZGAHBZAB01 for IL-1, and ZIBIBZAB02 for IL-6.

### 2.8. Determination of IKK*β* and NF-*κ*B p65 mRNA Expression in KCs

Total RNA was extracted from KCs using TRIZOL Reagent. The integrity of each RNA sample was evaluated by agarose gel electrophoresis, its purity and concentration were assayed, and then the total RNA was reverse transcribed to cDNA. Using the gene sequences for IKK*β*, NF-*κ*B p65, and glyceraldehyde-3-phosphate dehydrogenase (GAPDH) in Genebank, the primers were designed and synthesized by Shanghai Generay Biological Engineering Co., Ltd. GAPDH was used as the reference gene. The reaction conditions were as follows: ① predenaturation for 10 s at 95°C, ② denaturation for 5 s at 95°C, ③ GAPDH 55°C, IKK*β* 60°C, NF-*κ*B 62°C, renaturation for 20 s, ④ extension for 40 s at 60°C, ②–④ being repeated 39 times, and ⑤ analysis of the solubility curve, 72°C to 95°C for 5–10 s. After the reaction was finished, the results were analyzed using Opticon Monitor 3.1 software, and the 2^−ΔΔCt^  formula was used for relative quantification (Table 1).

### 2.9. Analysis of IKK*β*, p-IKK*β*, and NF-*κ*B p65 Protein Expression in KCs

The IKK*β*, p-IKK*β*, NF-*κ*B p65, and GAPDH proteins in KCs were measured by Western blotting. GAPDH was used as an internal control. KCs were lysed in RIPA lysis buffer and centrifuged at 12000 rpm for 5 min at 4°C, when the supernatants were collected. The concentration of supernatant protein was determined by a BCA protein assay. Protein preparations were subjected to 10% SDS-PAGE and transferred to a polyvinylidene difluoride (PVDF) membrane. After transfer, the membrane was blocked in 5% skim milk in Tris-Buffered Saline Tween-20 (TBST) and incubated overnight at 4°C with specific primary antibodies. The IKK*β* antibody (no. 0002), anti-phosphor-IKK*β* antibody (no. 0013), NF-*κ*B p65 antibody (no. 0006), and GAPDH antibody were purchased from Cell Signaling Technology (USA). Then, horseradish peroxidase (HRP) conjugated goat-anti-rabbit antibody was added and incubated at room temperature for 1 h. After being washed for three times in TBST, the PVDF membrane was put into developer and exposed to X-ray film. The films were scanned and analyzed by a gel image processing system.

### 2.10. Statistical Analysis

Statistical analyses were performed by using SPSS 13.0. The values were presented as the mean ± standard. One-way analysis of variance (one-way ANOVA) and Tukey's test were used to determine the statistical significance of the differences. *P* values less than 0.05 were considered significant.

## 3. Results

### 3.1. Histopathological Changes

**Figure 1:**
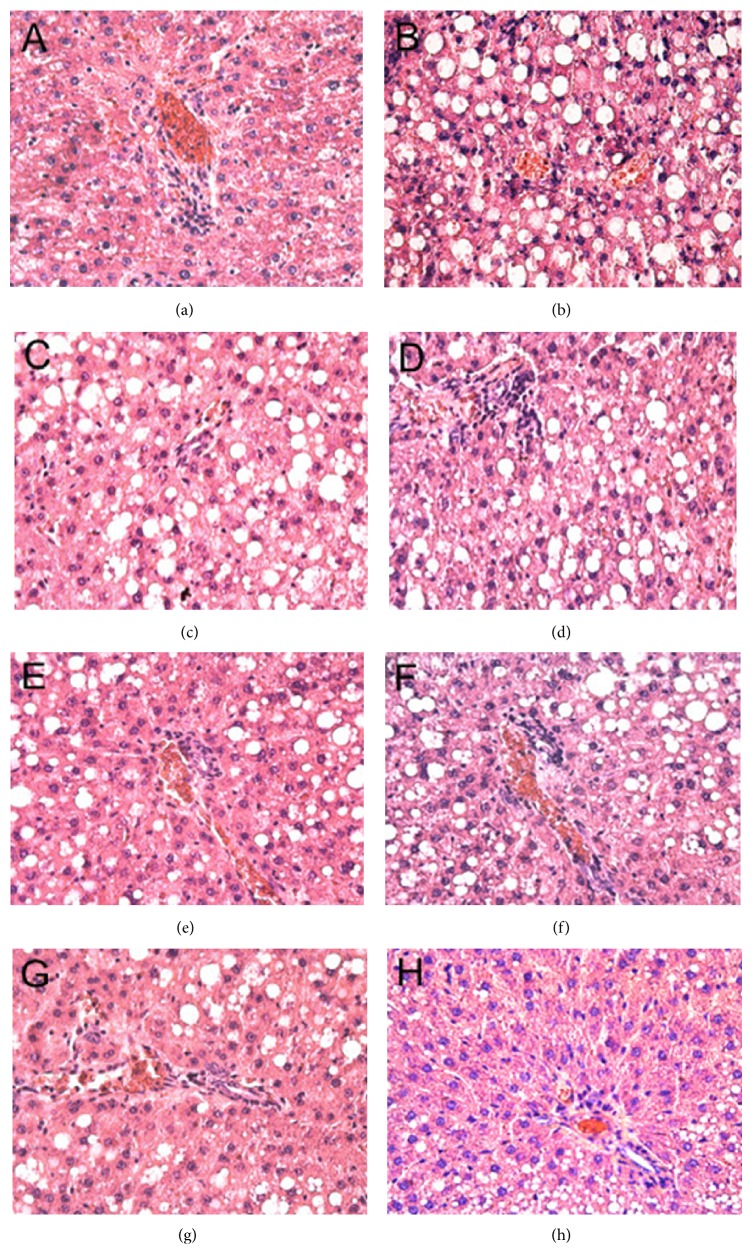
Histological changes of liver sections in different groups (HE stain ×200). (a) Normal group, (b) model group, (c) L-SLG, (d) H-SLG, (e) L-ISG, (f) H-ISG, (g) L-IG, and (h) H-IG.

Sections were stained with H&E staining. As shown in Figure 1, hepatocyte nuclei were blue, and cytoplasm was uniformly red-stained, with less or no adipose hollow space. The hepatic lobule and liver rope both had clear structures and regular arrangement. There was no point necrosis or soakage of inflammatory cell soakage. In the model group, the central vein and portal area appeared as a diffuse adipose hollow space. Hepatocytes had obvious tumefaction or enlargement or even ballooning. Substantial adipose hollow space was observed in the cytoplasm. Some small hollow spaces were converted into a larger space. The narrow hepatic sinusoidal and disorder liver rope were observed. It was difficult to find the normal hepatocytes. Inflammatory infiltrates were found in the hepatic lobule and portal areas are scant. Compared with the model group, the structure arrangement, morphological features, macrovesicular lipid droplets, ballooning, inflammatory infiltrates, and spots necrosis improved by various degrees in all drug therapy groups. H-IG had the most significant impact on liver tissue histopathology.

### 3.2. Changes of ALT, AST, and AST/ALT in Serum

**Figure 2:**
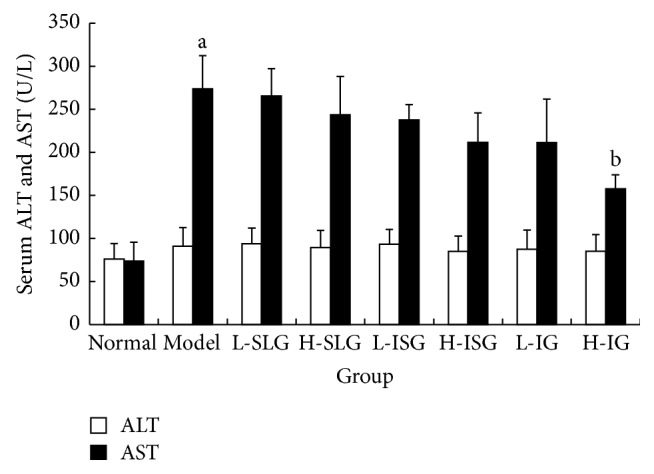
Levels of ALT and AST in serum were determined. Rats were fed with normal chow diet or HFD with or without CSS and SLBZS for 26 weeks. The values were expressed as mean ± S.E.M. 9 rats per group. ^a^ *P* < 0.01 versus normal group; ^b^ *P* < 0.01 versus model group.

**Figure 3:**
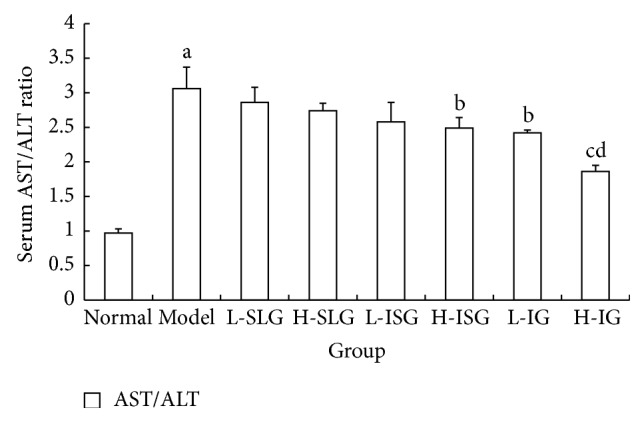
AST/ALT ratio in serum was computed. Rats were fed with normal chow diet or HFD with or without CSS and SLBZS for 26 weeks. The values were expressed as mean ± S.E.M. 9 rats per group. ^a^ *P* < 0.01 versus normal group; ^b^ *P* < 0.05, ^c^ *P* < 0.01 versus model AST/ALT; ^d^ *P* < 0.01 versus L-SLG, H-SLG, L-ISG, H-IS, and L-IG.

The ALT results showed no differences between the normal and model groups, and there were no obvious differences in the serum ALT levels of the treatment groups (*P* > 0.05). The serum AST level and AST/ALT ratio of the model group were obviously increased (*P* < 0.01) compared with normal group. Compared with model group, the H-IG group had a significantly decreased level of AST (*P* < 0.05). The AST/ALT ratios of the H-SG, H-IG, and L-IG were all decreased (*P* < 0.01, *P* < 0.05), as shown in Figures 2 and 3.

### 3.3. The Population, Purity, and Viability of KCs

**Figure 4:**
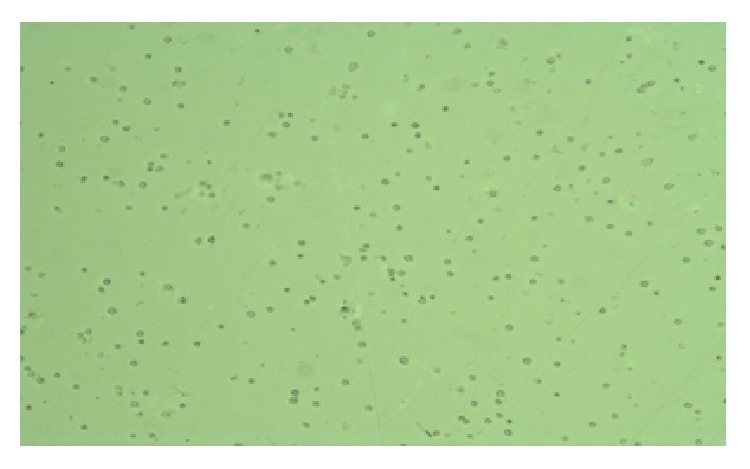
Isolated rat KCs cultured for 3 h (×100).

**Figure 5:**
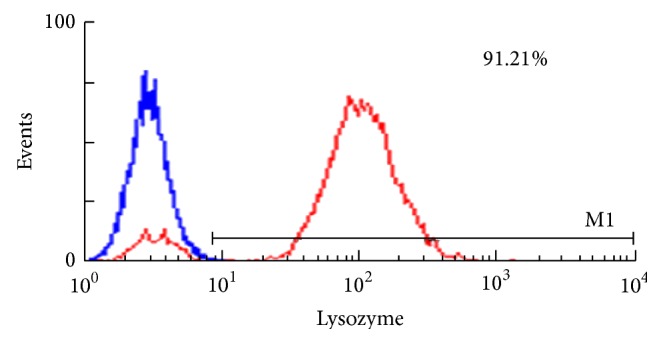
The purity of KCs identified by flow cytometer. Isolated rat KCs positive for lysozyme were more than 91.21%.

KCs were isolated from 6 rats in each group by collagenase perfusion as described. After 3 h incubation at 37°C, the cells were washed 3 times with PBS and nonadherent cells were washed off. The KCs viability was >95% (as tested by trypan blue dye exclusion). The purity of KCs was 91.21% (as assessed by flow cytometry method using a Lysozyme antibody), as shown in Figures 4 and 5.

### 3.4. Changes of Inflammatory Cytokines in KCs

**Figure 6:**
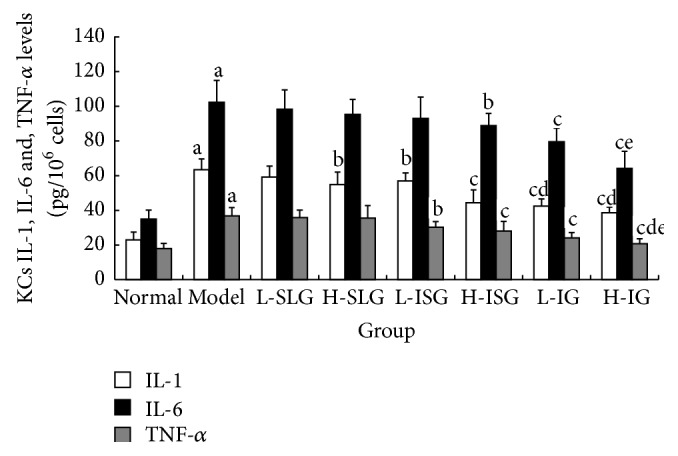
Related inflammatory cytokines of IL-1, IL-6, and TNF-*α* in KCs were determined by ELISA. Rats were fed with normal chow diet or HFD with or without CSS and SLBZS for 26 weeks. The values were expressed as mean ± S.E.M. 6 rats per group. ^a^ *P* < 0.01 versus normal group; ^b^ *P* < 0.05, ^c^ *P* < 0.01 versus model group; ^d^ *P* < 0.01 compared with H-SLG; ^e^ *P* < 0.01 compared with L-ISG.

Higher levels of TNF-*α*, IL-1, and IL-6 were observed in KCs of the model group compared with those of the normal group (*P* < 0.01). Compared with the model group, the H-ISG, H-IG, and L-IG groups showed significant decreases in TNF-*α* and IL-6 (*P* < 0.01 or *P* < 0.05), and the H-ISG and H-IG had clearly lower levels of IL-1 (*P* < 0.01 or *P* < 0.05). The results showed that the high dose of both the invigorating spleen recipe and integrated recipes reduced the TNF-*α*, IL-1, and IL-6 levels of liver inflammatory cytokine in rats with NASH induced by HFD, as shown in Figure 6.

### 3.5. The IKK*β* and NF-*κ*B Expression in KCs

Compared with the normal rats, the levels of IKK*β* and NF-*κ*B mRNA in KCs of the model group increased 20.56-fold and 16.29-fold, (*P* < 0.01). The H-CG, H-SG, H-IG, and L-IG treated animals had lower expression levels of IKK*β* and NF-*κ*B mRNA than the model rats (*P* < 0.01, *P* < 0.05). The expression levels of IKK*β* and NF-*κ*B mRNA in the H-IG were obviously lower than those of the H-SG and L-IG (*P* < 0.05), as shown in Table 2.

### 3.6. Expression of IKK*β*, p- IKK*β*, and NF-*κ*B p65 Proteins in KCs

**Figure 7:**
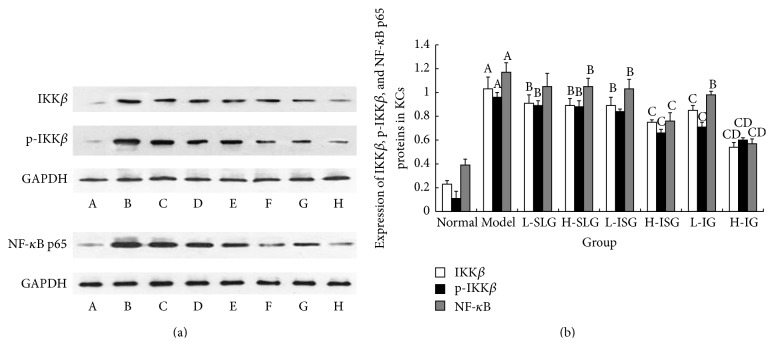
(a) Western blot of IKK*β*, p-IKK*β*, and NF-*κ*B p65 proteins in KCs. A: normal group; B: model group; C: L-SLG; D: H-SLG; E: L-ISG; F: H-ISG; G: L-IG; H: H-IG. (b) Expression of IKK*β*, p-IKK*β*, and NF-*κ*B p65 proteins in KCs. Rats were fed with normal chow diet or HFD with or without CSS and SLBZS for 26 weeks. The values were expressed as mean ± S.E.M. 6 rats per group. ^A^ *P* < 0.01 versus normal group; ^B^ *P* < 0.05, ^C^ *P* < 0.01 versus model group; ^D^ *P* < 0.01 versus L-SLG, H-SLG, L-ISG, H-IS, and L-IG.

To explore the mechanism of the anti-inflammatory effect of soothing liver and invigorating spleen recipes in the KCs of NASH rats, we assayed three important proteins, IKK*β*, p-IKK*β*, and NF-*κ*B, which participate importantly in the NF-*κ*B p65 signaling pathway to an inflammatory response, as shown in Figure 7(a). The expression levels of IKK*β*, p- IKK*β*, and NF-*κ*B p65 in the model group were significantly higher than those in the normal control group (*P* < 0.01, Figure 7(b)). Compared to the model group, the expression levels of IKK*β*, p- IKK*β*, and NF-*κ*B p65 were reduced in all treatment groups (*P* < 0.01, *P* < 0.05). We found that IKK*β*, p-IKK*β*, and NF-*κ*B p65 protein expression was inhibited in the H-IG more than in the H-CG, L-CG, H-SG, and L-SG (Figure 7(b)).

## 4. Discussion

In our previous study, a rat model of NASH was established using 16 weeks of HFD, so as to resemble the pathogenesis of NASH in humans more closely. In this study, we showed by H&E staining that NASH was successfully established. In the model group, the central vein and portal areas appeared as diffuse adipose hollow spaces. The hepatocytes had obvious tumefaction, enlargement, or even ballooning. The H&E staining also suggested that the different treatments affected the NAFLD to different degrees, with the H-IG superior to other groups.

Past studies indicated that the ALT and AST are the most useful tools for diagnosis of the chronic liver disease. Beyond these tools, the ALT/AST ratio is a prognostic parameter in patients with liver injury [16]. If hepatocytes are badly damaged and their cytoplasm and mitochondria are destroyed, AST is released into the blood and its level increases more than that of ALT, so the AST/ALT ratio increases [17]. The AST/ALT ratio has therefore become an important index for the diagnosis of NAFLD [18]. In this study, the serum AST levels and AST/ALT ratios were obviously increased (*P* < 0.05). We speculate that liver injury already exists in NAFLD rats. Combining the H&E staining and liver function tests, we can see that the liver has sustained serious damage from inflammation. We now show that a high dose of integrated recipes protects against liver injury and moderates NASH progression (*P* < 0.05). TCM theory attributes the abnormalities in the 26 weeks' HFD-induced NASH rats to liver stagnation and spleen deficiency. Therefore, the effects of the soothing liver recipe and the invigorating spleen recipe were superior to other classical formulas.

KCs are resident hepatic macrophages that account for 80–90% of the total number of fixed tissue macrophages of the body [19]. KCs eliminate and detoxify microorganisms, endotoxins, and degenerated cells, as well as possessing other functions [20]. Therefore, KCs play an important role in liver physiological homeostasis and are intimately involved in the liver's response to infection, toxins, and various other stresses through the expression and secretion of soluble inflammatory mediators [21, 22]. KCs are associated with the proinflammatory response and produce associated cytokines such as IL-1*β*, IL-12, IL-23, and TNF-*α*. Cytokines act as protective mediators for the recovery of normal liver function. However, excessive activation of KCs may aggravate liver damage [23, 24]. According to the two-hit hypothesis, the second hit is an exacerbating factor such as an inflammatory cytokine. Previous studies show that inflammatory KCs play a key role in NASH [25]. In this paper, the expression of IL-6 and TNF-*α* was significantly increased in KC supernatants from the model group (*P* < 0.01) while the levels in the supernatants of the low- and high-dose integrated recipes were significantly lower than the other groups (*P* < 0.01, *P* < 0.05). These findings suggest that increases in liver TNF-*α*, IL-1, and IL-6 may aggravate hepatic inflammation, necrosis, and fibrosis and that the high dose of the invigorating spleen and integrated recipes may have a favorable effect on the inflammatory reaction in the steatotic liver.

As everyone knows, excessive inflammatory cytokines such as TNF-*α*, IL-1, and/or IL-6 exacerbated liver injury and promoted NASH progression in different ways [26]. The mechanisms involved remain unclear, though many reports implicate the NF-*κ*B signal pathway. To elucidate the regulatory mechanisms in the NF-*κ*B signaling pathway and the anti-inflammatory effects of the soothing liver and invigorating spleen recipes further, we examined the expression and levels of several proteins closely related to the signal transduction in the NF-*κ*B pathway of KCs from NASH rats [27].

The IKK*β*-NF-*κ*B p65 signaling pathway is an important regulator of inflammatory gene transcription involved in many chronic inflammatory diseases. In NASH, different activators of the IKKs-I*κ*B-NF-*κ*B p65 signaling pathway in KCs regulate the synthesis of downstream inflammatory mediators. Possible pathways are reviewed in and include [28–31] (1) LPS captured by LPS-binding protein (LBP), with the LPS-LBP complex then interacting with the membrane form of CD14 on the surface of KCs. TLR4 serves as the LPS receptor and binds MyD88 to activate IRAK-1 or IRAK-4, leading to downstream activation of the IKKs-I Kb-NF-*κ*B signaling pathway. (2) The TNF receptor combined with its related apoptosis structural domain protein TRADD interacts with the TNFR-2 pathway through ubiquitinated receptor interacting protein RIP and finally forms the RNFR complex with IKK*γ*, which leads to activation of the IKKs-I*κ*B-NF-*κ*B signaling pathway. During hepatic steatosis, inflammation, and fibrosis, hepatic NF-*κ*B is highly expressed, though IKK*β*/NF-*κ*B pathway activation is inhibited. These findings suggest that it may be possible to delay the occurrence of inflammation and liver steatosis and insulin resistance (IR). (3) A previous study showed that, in IRF3 gene-knockout mice, activation of the IKK*β*/NF-*κ*B signaling pathway caused severe inflammation of the liver IR and fatty degeneration.

Traditional Chinese medicine has received increasing attention as an alternative source of treatments for a variety of diseases [32]. According to TCM theory, NAFLD belongs to the Gan-Pi and Gan-Zhu. Epidemiological researches showed that the syndrome of stagnation of liver qi and spleen deficiency is one of the most common syndromes of NAFLD in china, and the proportion is 34.7%. This study suggested the clinical characteristics of Chinese NAFLD population in contemporary [33]. Therefore, the syndrome of stagnation of liver qi and spleen deficiency has become the most important syndrome in expert consensus document of NAFLD [34]. According to Chinese medicinal chemistry, the principal active components of Senlinbaizhu Powder and Chaihushugan Powder include: ferulic acid, ginsenoside, paeoniflorin, naringin, hesperidin, meranzin hydrate, neohesperidin, albiflorin, and atractylenolide, together with other drug ingredients [35, 36]. At the same time, Chinese medicinal pharmacology has demonstrated that Chaihu-Shugan-San and Shen-ling-bai-zhu-San have inhibitory activities on oxidative stress [37], lipid peroxidation, and inflammatory reactions [38]. Beyond that, naringin, hesperidin, ferulic acid, and other active ingredients have some anti-inflammatory effects [36, 37]. Our results show that soothing liver and invigorating spleen recipes can protect the liver from inflammatory injury caused by an HFD, that the release of IL-1, IL-6, and TNF-*α* was significantly reduced, and that the downregulation of IL-1, IL-6, and TNF-*α* might be due to different degrees of inhibitory expression of IKK*β*, p-IKK*β*, and NF-*κ*B. A previous study showed that the soothing liver and invigorating spleen recipes could regulate the expression and activation of the interacting protein of IKK*β*-NF-*κ*B signaling pathway of KCs in NASH rats, reducing the release of inflammatory mediators (IL-6, TNF-*α*, and IL-1) in KCs and, ultimately, ameliorating inflammatory damage. Consequently, a combination of the soothing liver and invigorating spleen recipes could have a significant anti-inflammatory effect, which might be closely related to their effects on the NF-*κ*Bp65 signaling pathway.

## 5. Conclusion

In conclusion, this study showed that the release of inflammatory factors such as IL-1, TNF-*α*, and IL-6 by KCs was significantly increased by a HFD and that the IKK*β*-NF-*κ*Bp65 signaling pathway maybe the effective target for the soothing liver and invigorating spleen recipes.

## Figures and Tables

**Table 1:** Primer sequences, annealing temperatures, and length of products in real-time PCR.

Gene	Forward	Backward
GAPDH	GATCCCGCTAACATCAAATG	GAGGGAGTTGTCATATTTCTC
IKK*β*	GAGAAGAAAGTGCGGGTGATTTACT	GAGCCTCACCACCTCTTCTACTTT
NF-*κ*B	GTGGGCAAGCACTGTGAGGA	TCATCCGTGCTTCCAGTGTTTC

**Table 2:** Expression of IKK*β* and NF-*κ*B mRNA in KCs ($\overset{-}{x}\pm s$, *n* = 6).

	2^−ΔΔCT^ IKK*β* Rel. to control	2^−ΔΔCT^ NF-*κ*B p65Rel. to control
Normal	1 (0.25–4.09)	1 (0.39–2.65)
Model	20.56 (4.56–34.05)^a^	16.29 (2.77–48.16)^a^
L-SLG	17.99 (1.89–30.69)	14.43 (2.33–31.12)
H-SLG	14.34 (3.51–23.43)^b^	10.39 (2.15–22.31)^b^
L-ISG	15.63 (7.31–28.05)	12.07 (7.06–15.78)
H-ISG	10.32 (3.83–23.91)^c^	9.15 (4.43–27.10)^c^
L-IG	12.09 (4.75–20.11)^b^	9.85 (2.56–18.90)^b^
H-IG	6.55 (1.80–14.83)^cd^	4.76 (0.55–12.47)^cd^

Expression of IKK*β* and NF-*κ*B mRNA in KCs was determined by Q-PCR. Rats were fed with normal chow diet or HFD with or without CSS and SLBZS for 26 weeks. The values were expressed as mean ± S.E.M. of 6 rats per group. ^a^ *P* < 0.01 versus normal group; ^b^ *P* < 0.05, ^c^ *P* < 0.01 versus model group, ^d^ *P* < 0.01 versus H-ISG and L-IG group.
